# Obesity associated with a novel mitochondrial tRNA^Cys^ 5802A>G mutation in a Chinese family

**DOI:** 10.1042/BSR20192153

**Published:** 2020-01-06

**Authors:** Jinling Wang, Ningning Zhao, Xiaoting Mao, Feilong Meng, Ke Huang, Guanping Dong, Yanchun Ji, JunFen Fu

**Affiliations:** 1 Department of Endocrinology, The Children’s Hospital, Zhejiang University School of Medicine, National Clinical Research Center For Child Health, Hangzhou, China; 2 Division of Medical Genetics and Genomics, The Children’s Hospital, Zhejiang University School of Medicine, National Clinical Research Center For Child Health, Hangzhou, Zhejiang 310058, China; 3 Institute of Genetics, Zhejiang University, Hangzhou, Zhejiang 310058, China

**Keywords:** Children, Chinese, mitochondrial tRNA, mutation, obesity

## Abstract

A Chinese family with matrilineally inherited obesity was assessed and its clinical, genetic, and molecular profiling was conducted. Obesity was observed in matrilineal relatives (3 out of 7) of a single generation (of 3 alive generations) in this family. On pedigree analysis and sequencing of their mitochondrial DNA (mtDNA), a novel homoplasmic mutation of the mitochondrial tRNA^Cys^ gene (5802A>G) was identified in these individuals. This mutation correlated with a destabilized conserved base pair in this tRNA anticodon stem. Position 30 is known to be crucial for carrying out effective codon recognition and stability of tRNA. In accordance with the importance of this conserved site, we observed that the predicted structure of tRNA^Cys^ with the mutation was noticeably remodeled in a molecular dynamics simulation when compared with the isoform of the wild-type. All other 46 mutations observed in the individual’s mtDNA were known variants belonging to haplogroup D4. Thus, this is the first report that provides evidence of the association between a mutation in tRNA and an enhanced risk of maternally transmissible obesity, offering more insights into obesity and its underlying nature.

## Introduction

A major global health challenge is obesity, which over the last 40 years has witnessed more than 10-fold increase childhood obesity children [1,2]. Obesity is caused due to several factors that are not yet fully understood [3]. Moreover, obesity in childhood can often be complex accompanied by underlying cardiovascular disease, hypertension, abnormal puberty, or Type 2 diabetes [3,4]. While single gene mutations can mediate obesity, it can occur as a result of interaction of genetic and environmental factors. To date, nuclear gene mutations linked to obesity such as *MC4R, BDNF, FTO*, and *PCKS1*, have been detected [5]. Nonetheless, the association of mutations in mitochondrial DNA (mtDNA) and the onset of childhood obesity still remains to be unraveled. Several mutations in the mitochondrial tRNA genes have been found to be linked to diabetes and metabolic disorders. It was indicated that mutations in mtDNA may be associated with obesity. For instance, the tRNA^Thr^ mutation m.10003T>C, as well as the tRNA^Glu^ mutations m.14709T>C, and m.14692A>G have been associated with such disease [6–8].

Therefore, their association with other metabolic disorders indicates that mitochondrial tRNA mutation may potentially be associated with enhanced childhood obesity risk. Thus, we therefore attempted to detect deleterious mutations throughout systematic sequencing of the mitochondrial genomes of obese children in China. In this investigation, we found that a novel m.5802A>G mutation in the tRNA^Cys^ gene located in the H (heavy) strand of the mtDNA may be potentially a risk factor in a obese pedigree. For estimating a significant correlation of mutations with obesity, we mainly focused on the mutations that are evolutionarily conserved in 16 other vertebrate species, particularly present in <1% of non-obese controls, and have characteristic secondary structural elements of the tRNA including loops, hairpins, or anticodon loops, the stem region, because mutations in these regions may be more likely to affect the translational activity by altering interactions between codons and anticodons, or tRNA stability [9,10]. Moreover, we allocated the identified mtDNAs of the subjects to specific Asian mtDNA haplogroups as per nomenclature of mtDNA haplogroups [11]. Finally, we computed the structural analysis and simulations of molecular dynamics in the mitochondrial tRNA gene.

## Subjects and methods

### Subjects

An obese Chinese boy from Han province (9.3 years old) from our endocrinology department with uncontrolled weight-gaining over the last 4 years was the subject of study. A BMI more the 95th percentile for their age and sex was defined as obesity and above 99 percentiles as severe obesity as per Chinese Working Group on Obesity and CDC childhood obesity guidelines [12,13]. The present study complied with the Declaration of Helsinki. The Ethic Committees of the Children’s Hospital, School of Medicine, Zhejiang University approved the methodologies for obtaining blood samples, and clinical evaluations from all participating family members, including their informed, written consent. A comprehensive history and physical examination for the participating individuals were assessed and underwent elaborate evaluation for personal as well as family medical histories of obesity and other clinical abnormalities. The control children (*n* = 106) were recruited from the same region and were used as control for screening the presence of variants.

### Clinical examinations

Physical examination and laboratory biochemical evaluation were carried out on the subjects. Participants wore light clothing and wore no shoes, and the measurements were taken to nearest 0.5 cm and 0.1 kg, respectively for height and weight. The formula of weight (kg) per by height (m^2^) was denoted as BMI. Blood pressure was measured twice when the subject sat in a quiet position, and the average for the right arm was recorded.

The biochemical assessment of routine parameters was done, including total cholesterol, fasting triglycerides, LDL, HDL, ALT, and AST. A standard oral glucose (75 g) tolerance test was done by recording the levels of glucose and insulin. Using a convex 3.5–5.0 MHz probe, and the equipment (GE, LOGIC 500), liver B-Ultrasound examination was carried out and blinded to laboratory values on the same equipment (GE, LOGIC 500) using a convex 3.5-5.5 MHz probe.

### Assessment of mutations in mtDNA

Whole genomic DNA was extracted with the puregene DNA isolation kit (Qiagen) that was used to extract whole gnomic DNA, and the entire genome of mitochondrial was amplified total of three pooled reactions by PCR as 99 separate fragments, using primers for the light and heavy stranded DNA. Then the DNA was purified using magnetic beads and a library was made for directly sequenced on a sequencer using a next-generation sequencing reaction kit (MiniSeq, Illumina). To identify mutations within the obtained genome, the consensus Cambridge sequence (GenBank accession number: NC_012920) was used as a reference [14].

To identify the m.5802A>G mutation in mitochondrial L-strand region of the mitochondrial tRNA^Cys^ gene, the 5238-6050 region was amplified using: F:5′-CTAACC GGCTTTTTGCCC-3′ and R:5′-ACCTAGAAGGTTGCCTGGCT-3′, as described previously [14] and analyzed as mentioned previously [15].

### Analyses of mitochondrial haplogroups

Asian mitochondrial haplogroups were the identification of asian mitochondrial haplogroups that were performed as per the conventions of mitochondrial haplogroup nomenclature using an online tool (http://www.mitotool.org/genomeRSRS.html) [11,16].

### Structural analysis and simulations of molecular dynamics

The secondary structural elements of tRNA were identified as per secondary structures published previously [17].

To identify the corresponding coordinates to the loop (ASL) and to the wild-type tRNA^Cys^ anticodon stem, the reference used was the Sus scrofa mitochondrial tRNA (Protein Data Bank entry 5AJ3). The nucleoside bases per the mitochondrial tRNA^Cys^ sequence were substituted by applying the Chimera program [18]. Similarly, the A30C mutation coordinates were generated using Chimera, on the basis of wild-type tRNA^Cys^. NaCl (50 mM) was added as a solvent, which besides Na^+^ or Cl^−^ neutralization, yielded a wild-type ASL system and a mutant system consisting of 8996 atoms and 9015 atoms, respectively.

The simulation for MD was conducted using the Amber14 program [19]. Trajectories for MD were propagated by a shaking algorithm for each hydrogen with a non-bonded cut off every 0.02 fs [20]. Unfavorable contacts were relieved by solvent equilibration, equilibrating with a periodic boundary condition at 1 bar and 300 K in the NPT ensemble and by energy minimization. Initially, positional restraints were thrust on the all ALS atoms for 500PS. Production simulations were carried out after equilibration, at 2 fs time intervals for up to 100 ns in total.

### Phylogenetic analysis

Interspecific analysis was carried out using the mitochondrial tRNA sequences of 16 vertebrate species, as mentioned earlier [21]. A CI (conservation index) value was estimated by comparing the human tRNA gene nucleotides with those of the other 15 vertebrate species; CI was determined as the percentage of the assessed species having the human wild-type nucleotide at the specified position.

### Statistical analysis

For all statistical testing was done using SPSS v17.0 with (significance threshold *P* < 0.05). Fisher’s exact test was used to compare of tRNA mutations frequencies between control and children.

## Results

### Case study

**Figure 1:**
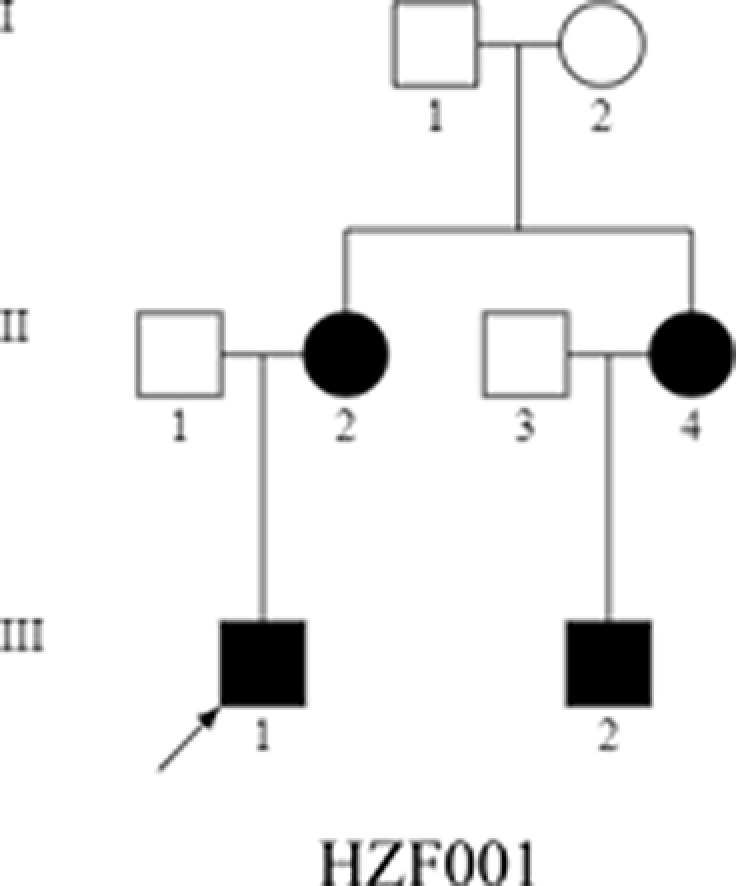
The pedigree for obesity Obese individuals are indicated by dark symbols.

A 9.3-year-old Han Chinese boy was the enrolled proband (III-1) in the investigation, who experienced constant weight gain over the last 4 years (Figure 1). Although there were no reports of other health problems prior to the obesity issue. As shown in Table 1, physical examination revealed thickened dark skin (acanthosis nigricans) on the neck and in the armpits with 40.5% body fat. The boy had a height of 154.0 cm, weight of 60.0 kg, the waist–hip ratio of 1.03, and blood pressure 109/75 mmHg, 25.3 kg/m^2^ BMI, categorized as severely obese. As per Tanner Staging, the subject was in B3 with penis length 6 cm and testicular volume 10 ml. The measurements of his mother (II-2) had BMI 25.7 kg/m^2^, height 165.0 cm, weight 70.0 kg and was overweight, while the father was not overweight with BMI 20.7 kg/m^2^. His aunt (II-4) had BMI 25.8 kg/m^2^ and was overweight.

**Table 1:** Summary of clinical data for affected matrilineal relatives of a Chinese families with obesity

Subjects	Age (years)	Sex	Height (cm)	Weight (kg)	BMI (kg/m^2^)	Obese duration (years)
III-1	9.3	M	154	60	25.3	4
II-2	38.2	F	165	70	25.7	>10.0
II-4	35.5	F	165	70	25.7	>10.0
III-2	6.3	M	128	40	24.4	1

Insulin resistance in the proband was observed through biochemical assessment, with insulin >300 uIU/ml without Type 2 diabetes mellitus, as per OGTT results. The liver function was normal, but with NALFD as determined by B-ultrasound. The lipid profile showed all parameters in range including trigleceride 1.64 mmol/l, cholesterol 3.76 mmol/l, HDL 1.16 mmol/l, LDL 2.39 mmol/l, Non-HDL 2.60 mmol/l. Finally, the participant was diagnosed with severe obesity, having metabolic disorders including acanthosis nigricans, insulin resistance, hyperuricemia, and NALFD.

### Analysis of mtDNA mutation

**Figure 2:**
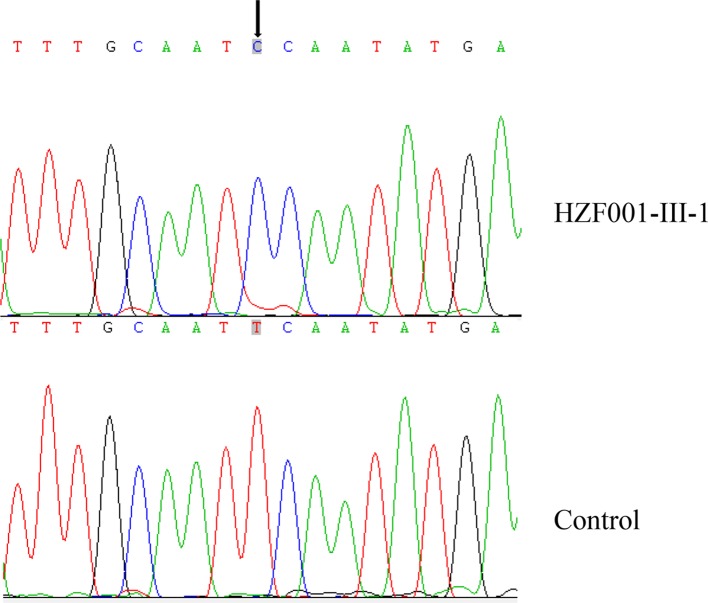
Detection of m.5802A>G in the tRNA The chromatograms tRNA sequence from proband and control individuals. The arrow points to the identified mutation site.

Complete mitochondrial genome of this subject was sequenced to assess the role of genetics in the obesity of this subject. When this sequence was compared with the Cambridge consensus sequence, 47-point variants were detected which coincided with the Eastern Asian haplogroup D4 (Table 2). Among these mutations, 8 were that of the D-loop region, 3 were of 12S rRNA, 2 was of 16S rRNA, and a novel homoplasmic m.5802A>G mutation was observed affecting the gene for tRNA^Cys^ (Figure 2). The remaining included 20 already known silent mutations, and 13 were missense mutations in protein coding regions which were: the m.8414C>T(Leu17Phe) in the ATP8 gene, m.8701A>G (Thr59Ala) m.8830C>A(Leu102Met) and m.8860A>G(Thr112Ala) in the ATP6 gene, the m.10398A>G(Thr114Ala) in the ND3 gene, the m.13748A>G(Asn471Ser), m.13753T>C(Ser473Pro), m.13754C>T(Ser473Phe), m.13759G>A(Ala475Thr) and m.13775C>T(Thr480Met) in the ND5 gene, the m.14766C>T(Thr7Ile), m.15236A>G(Ile164Val) and m.15326A>G(Thr194Ala) in the CYTB gene. These variants were evaluated for the pathogenicity using the following criteria: (1) present in <1% of the control subjects; (2) evolutional conservation >75%; (3) potential structural alterations [8]. We next carried out a phylogenetic analysis to evaluate the extent of conservation of these different mutated sites in humans compared with 16 other primate species. A 100% CI value for the m.5802A>G mutation was found, with no clear evolutionary conservation for other mutations identified (Table 3). This proband subject (HZF001) was also found to belong to the Eastern Asian halpogroup D4 as per the standard haplogroup designation nomenclature.

**Table 2:** mtDNA variants in a Chinese family with obesity

Gene	Position	Replacement	CRS	HZF001	Previously reported
D-loop	73	A-G	A	G	Yes
	194	C-T	C	T	Yes
	263	A-G	A	G	Yes
	310	C-CC	C	CC	Yes
	16093	T-C	T	C	Yes
	16223	C-T	C	T	Yes
	16362	T-C	T	C	Yes
	16519	T-C	T	C	Yes
*MT-RNR1*	750	A-G	A	G	Yes
	1382	A-C	A	C	Yes
	1438	A-G	A	G	Yes
*MT-RNR2*	2706	A-G	A	G	Yes
	3010	G-A	G	A	Yes
*MT-ND2*	4721	A-G	A	G	Yes
	4769	A-G	A	G	Yes
	4883	C-T	C	T	Yes
	5178	C-T	C	T	Yes
*MT-TC*	5802	T-C	T	C	No
*MT-COX1*	7028	C-T	C	T	Yes
	7076	A-G	A	G	Yes
*MT-COX2*	8020	G-A	G	A	Yes
*MT-ATP8*	8414	C-T( Leu17Phe)	C	T	Yes
*MT-ATP6*	8701	A-G(Thr59Ala)	A	G	Yes
	8830	C-A(Leu102Met)	C	A	Yes
	8860	A-G( Thr112Ala)	A	G	Yes
	8964	C-T	C	T	Yes
*MT-COX3*	9296	C-T	C	T	Yes
	9540	T-C	T	C	Yes
	9932	G-A	G	A	Yes
*MT-ND3*	10398	A-G(Thr114Ala)	A	G	Yes
	10400	C-T	C	T	Yes
*MT-ND4*	10873	T-C	T	C	Yes
	11719	G-A	G	A	Yes
*MT-ND5*	12705	C-T	C	T	Yes
	13748	A-G( Asn471Ser)	A	G	Yes
	13753	T-C(Ser473Pro)	T	C	Yes
	13754	C-T( Ser473Phe)	C	T	Yes
	13759	G-A(Ala475Thr)	G	A	Yes
	13775	C-T( Thr480Met)	C	T	Yes
	13776	A-G	A	G	Yes
*MT-ND6*	14668	C-T	C	T	Yes
*MT-CYTB*	14766	C-T( Thr7Ile)	C	T	Yes
	14783	T-C	T	C	Yes
	15043	G-A	G	A	Yes
	15236	A-G(Ile164Val)	A	G	Yes
	15301	G-A	G	A	Yes
	15326	A-G( Thr194Ala)	A	G	Yes

**Table 3:** Alignment of the MT–TC gene from 17 different species

Organism	Acc-stem		D-stem	D-loop	D-stem		Ac-stem	Anticd-loop	Ac-stem	V-region	T-stem	T-loop	T-stem	Acc-stem	
	1	8	10		22	26	27	32	39	44	49		61	66	73
Cebus albifrons	AGCCCTG	AG	GTGA	ACTG	TCAT	G	TTGAA	CTGCAAA	TTCAA	AGAA	GCAGC	TTCAAT	GCTGC	CGGGGCT	T
Cercopithecus aethiops	AGCCCCG	AG	GTGA	TTT	TCAT	G	TTAAA	TTGCAAG	TTTAA	AGGA	GCAGT	TTTGAGTT	TCTGC	CGGGGCT	T
Colobus guereza	AGTCCCG	AG	GTGA	TTT	TCAT	G	TTGAA	TTGCAAA	TTCAA	AGGA	GCAGC	TTAAGACC	TCTGC	CGGGGCT	T
Gorilla gorilla	AGCTCCG	AG	GTGA	ATT	TCAT	A	TTGAA	TTGCAAA	TTCGA	AGAA	GCAGC	TTCAAA	CCTGC	CGGGGCT	T
Homo sapiens	AGCTCCG	AG	GTGA	TTT	TCAT	A	TTGAA	TTGCAAA	TTCGA	AGAA	GCAGC	TTCAAA	CCTGC	CGGGGCT	T
Hylobates lar	AGTCCCG	AA	GTGG	TTT	TCAC	G	TTGAA	TTGCAAA	TTCAA	AGGA	GCAGC	TTCAAT	CCTGC	CGGGGCT	T
Lemur catta	AGCCCTG	TA	GTGA	ATA	TCAC	G	TTGGA	TTGCAAA	TTCAA	AGAA	GCAGC	TTCAAT	TCTGC	CGGGGCT	T
Macaca mulatta	AGCCCCG	AG	GTGA	TTT	TCAT	G	TTGAA	TTGCAAG	TTCAA	AGGA	GCAGT	CTTAGAGTT	TCTGC	CGGGGCT	T
Macaca sylvanus	AGCTCCG	AG	GTGA	TTT	TCAT	G	TTGAA	TTGCAAA	TTCAA	AGGA	GCAGT	TCCAAAGTT	TCTGC	CGGGGCT	T
Nycticebus coucang	GGCCTCG	AG	GTGA	TAAA	TCAT	A	TTGAA	TTGCAAA	TTCAA	AGGA	GCAGC	TTCAAT	TCTGC	CGGGGCT	T
Pan paniscus	AGCTCTG	AG	GTGA	TTT	TCAT	A	TTGAA	TTGCAAA	TTCAA	AGAA	GCAGC	TTCAAA	CCTGC	CGGGGCT	T
Pan troglodytes	AGCTCTG	AG	GTGA	TTT	TCAT	A	TTGAA	TTGCAAA	TTCGA	AGAA	GCAGC	TTCAAA	CCTGC	CGGGGCT	T
Papio hamadryas	AGCCCCG	AG	GTGA	TTT	TCAC	A	TTGAA	TTGCAAG	TTCGA	AGGA	GCAGC	TTTAAGTT	TCTGC	CGGGGCT	T
Pongo pygmaeus	AGCCCTG	AG	GTGA	TTG	TCAT	G	TTGAA	TTGCAAA	TTCGA	AGGA	GCAGC	TTTAAGG	CCTGC	CGGGGCT	T
Pongo pygmaeus abelii	AGCCCCG	AG	GTGA	TTG	TCAT	G	TTGAA	TTGCAAA	TTCGA	AGGA	GCAGC	TTTAAGG	CCTGC	CGGGGCT	T
Tarsius bancanus	AGTCCTG	AA	GTGA	ATA	TCAT	A	TTGAA	TTGCAAA	TTCAA	AGAA	GCAGC	TTCAAT	TCTGC	CGGGACT	T
Trachypithecus obscurus	AGCCCCG	AG	GTGG	TTT	TCAT	G	TTGAA	TTGCAAA	TTCAA	AGGA	GCAGT	TAGATT	TCTGC	CGGGGCT	T

Position 30 is the location of the m.5802T>C mutation.

### Simulation of molecular dynamics

**Figure 3:**
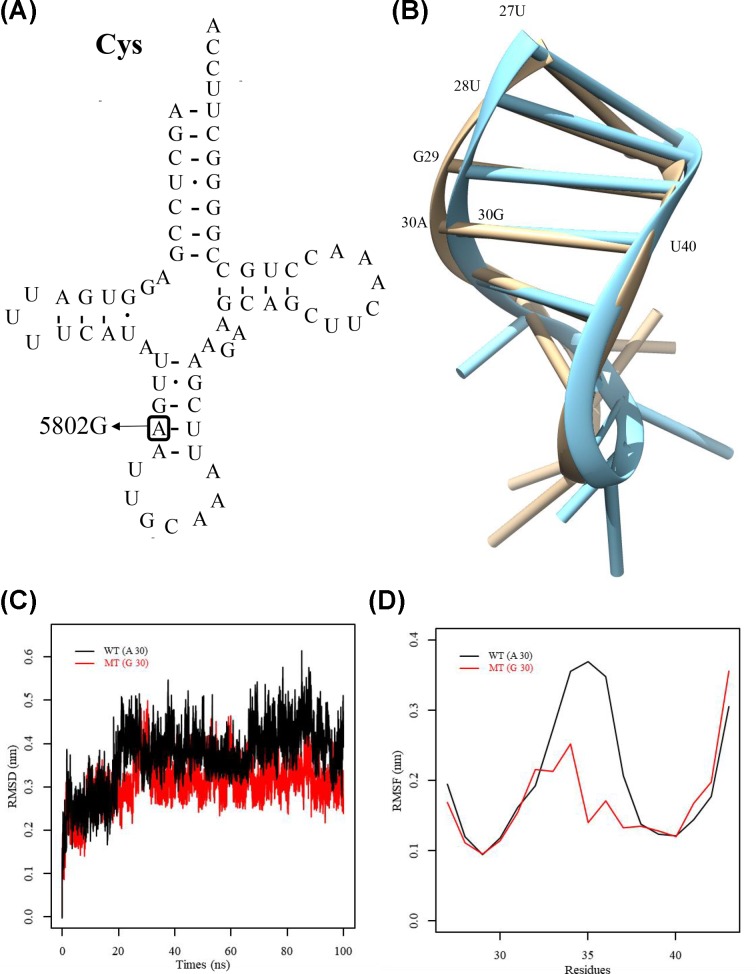
Structure of mitochondrial tRNA^Cys^ and MD simulations (**A**) The tRNA^Cys^ normally has a structure like that of a clover-leaf, and the identified mutation site is marked by an arrow. (**B**) The wild-type and mutated anticodon stem-loop simulated tertiary structure of this tRNA is shown (brown and blue, respectively). (**C**) RMSD (root mean square deviation) values with respect to time for the Cα atoms are mentioned for mutant and wild-type tRNA^Cys^ (red and black, respectively). (**D**) The RMSF curves for the mutant and wild-type isoforms (red and black, respectively) were constructed using the anticodon stem loop’s backbone atoms of tRNA^Cys^.

High evolutionary conservation was observed at the A30 site (Figure 3A,B), which may be important to the tRNA^Cys^ overall structural integrity, in agreement with its localization observed in the region of acceptor stem. Its structural importance was further confirmed by conducting a MD simulation assay to evaluate the role of this m.5802A>G mutation in the tRNA^Cys^ structural changes as the simulation approach has been confirmed as a means to evaluate the affect of disease-associated mutations on protein structure [22,23]. More substantial changes in the RMSD curve for the isoform of wild-type acceptor stem region in comparison with the mutated isoform over the simulation period was observed, indicating the structural impact of this mutation on the ASL region of this tRNA (Figure 3C). Furthermore, we carried out RMSF analysis on the two trajectories to analyze the mobility of ASL. As shown in Figure 3C, the RMSF values of A30 in wild-type ASL were much higher than that of the mutated isoform, further supporting that the mutant ASL was more stable than that of wild-type counterpart. These data strongly indicated that the 30G-40U base-pairing of anticodon stem of tRNA^Cys^ accounted for the more stability of the tRNA^Cys^ structure (Figure 3D).

## Discussion

We investigated here the clinical, genetic, and molecular findings from a Han Chinese family with symptoms of obesity, obesity across three generations in the matrilineal relatives of this pedigree, and obesity manifesting over a range of ages and initiating at 4 years in this proband individual. This obesity was seen to be transmitted in a matrilineally, indicating a potential mutation in the mtDNA as the underlying cause. Hence, we sequenced the mtDNA of the proband subject, and found 47 mutations in consistency with the Eastern Asian haplogroup D4 [11,12], of which were not conserved evolutionarily and are therefore not potentially linked to the disease. The remaining m.5802A>G homoplasmic mutation, however, affected a nucleotide (A30) with a greater degree of evolutionary conservation and is present within the tRNA^Cys^ acceptor stem and is important for maintaining the stability of tRNA [21]. Thus, this mutation probably affects the amino acid translation efficiency, impedes protein synthesis and induces mitochondrial dysfunction. Particularly, it may affect the expression of the compound enzyme that catalyzes mitochondrial oxidative phosphorylation initiates required for respiratory chain and enzyme activity. In response to aberrant oxidative phosphorylation, induction of a series of pathological alterations may occur, including oxygen free radicals production and a decline in the use of nitric oxide. The destabilization of base-paring hypothesized to occur due to this mutation is likely to alter the tRNA structure, as previously reported for other mutations including tRNA^Thr^ 15927G>A, tRNA^Ile^ 4300A>G, and tRNA^Leu(UUR)^ 3273T>C [22,24,25]. This hypothesis was further confirmed by an MD simulation, revealing that the m.5802A>G mutation changed the stability and mobility of tRNA secondary structure. Moreover, the m.5802A>G mutation perturbed the tRNA^Cys^ conformation, and correspond to the altered electrophoretic mobility of mutated tRNAs carrying the m.4435A>G, m.3253T>C and m.15927A>G mutations [22,26,27].

In this family, obesity started setting in at 9.3 years age. In contrast, the affected subjects carrying the m.5802A>G mutation suffered from being overweight or obese, like those exhibited in T2DM patients carrying m.10003T>C mutation [6]. Furthermore, a very low penetrance of obesity subjects was observed in this Chinese pedigree harboring the m.5802A>G mutation. Furthermore, this mutation (m.5802A>G) was not detected in the 106 Chinese control subjects. Therefore, the m.5802A>G mutation is itself insufficient to result in the clinical obesity because as observed through incomplete penetrance of obesity. These findings suggested that the phenotypic manifestation carrying the m.5802A>G mutation requires other modifying factors, including mitochondrial haplotype, nuclear background, and environmental factors. The mitochondrial haplotypes influence the expressivity and penetrance of obesity related to primary mtDNA mutations, although, other known mutations were not detected in this pedigree. In addition, in the family studied in this research, the mitochondrial genomes of the HZF001 pedigree categorized as the Eastern Asian haplogroup D4. However, data from only one family were analyzed, and the lack of a larger dataset cannot fully explain the role the mitochondrial haplotype.

Herein, we report a novel mitochondrial tRNA mutation that has a probable link to obesity in childhood in a Chinese population. Our results along with the observations of MD simulation indicate that the m.5802A>G mutation may be a possible inherited risk factor linked to the diagnosis of obesity. The results of the present study suggest that the m.5802A>G mutation may have relevance as a possible inherited risk factor for the development of obesity. However, the mild biochemical defects and the lower penetrance of obesity in this Chinese family suggested the involvement of other modifier factors in the pathogenesis of obesity. Further studies will be needed to definitively assess the relationship between mitochondrial dysfunction and the onset of obesity *in vivo*, in order to better understand how to manage genetic obesity risks in the global population.

## Abbreviations

ALT: alanine aminotransferase
AST: aspartate aminotransferase
BMI: body mass index
CI: conservation index
HDL: high-density lipoprotein
LDL: low-density lipoprotein
MD: molecular dynamics
NALFD: non-alcoholic fatty liver disease
OGTT: oral glucose tolerance test
RMSD: root mean square deviation
RMSF: root mean square fluctuation
